# Conduction block in myelinated axons induced by high-frequency (kHz) non-symmetric biphasic stimulation

**DOI:** 10.3389/fncom.2015.00086

**Published:** 2015-07-06

**Authors:** Shouguo Zhao, Guangning Yang, Jicheng Wang, James R. Roppolo, William C. de Groat, Changfeng Tai

**Affiliations:** ^1^Department of Urology, University of PittsburghPittsburgh, PA, USA; ^2^Department of Biomedical Engineering, Beijing Jiaotong UniversityBeijing, China; ^3^Department of Pharmacology and Chemical Biology, University of PittsburghPittsburgh, PA, USA

**Keywords:** nerve, block, simulation, high-frequency, model

## Abstract

This study used the Frankenhaeuser–Huxley axonal model to analyze the effects of non-symmetric waveforms on conduction block of myelinated axons induced by high-frequency (10–300 kHz) biphasic electrical stimulation. The results predict a monotonic relationship between block threshold and stimulation frequency for symmetric waveform and a non-monotonic relationship for non-symmetric waveforms. The symmetric waveform causes conduction block by constantly activating both sodium and potassium channels at frequencies of 20–300 kHz, while the non-symmetric waveforms share the same blocking mechanism from 20 kHz up to the peak threshold frequency. At the frequencies above the peak threshold frequency the non-symmetric waveforms block axonal conduction by either hyperpolarizing the membrane (if the positive pulse is longer) or depolarizing the membrane (if the negative pulse is longer). This simulation study further increases our understanding of conduction block in myelinated axons induced by high-frequency biphasic electrical stimulation, and can guide future animal experiments as well as optimize stimulation parameters that might be used for electrically induced nerve block in clinical applications.

## Introduction

High-frequency (kHz) biphasic electrical stimulation has recently been investigated extensively due to its potential clinical application to block peripheral nerve conduction (Nashold et al., 1982; Tai et al., 2004; Camilleri et al., 2009; Waataja et al., 2011). Although the mechanisms underlying this nerve block are still unclear (Zhang et al., 2006; Ackermann et al., 2011), previous animal studies of myelinated axons have shown that the block threshold intensity monotonically increases as the stimulation frequency increases up to 50 kHz (Bhadra and Kilgore, 2005; Gaunt and Prochazka, 2009; Joseph and Butera, 2009). Our recent computer simulation study of large (10–20 μm diameter) myelinated axons further indicates a monotonic increase in block threshold up to 100 kHz (Tai et al., 2011). However, recent animal studies (Joseph and Butera, 2009, 2011) revealed that this monotonic relationship does not hold in small unmyelinated axons where the block threshold current only increases with frequency up to about 12–15 kHz and then decreases as the stimulation frequency further increases. This discovery raises the question about what causes the difference of high-frequency block between myelinated and unmyelinated axons. Answering this question will help to understand the mechanisms underlying nerve conduction block induced by high-frequency biphasic electrical stimulation.

Due to the difficulties in recording ion channel activity in axonal nodes during high-frequency biphasic electrical stimulation, the mechanisms of nerve block have been mainly investigated by modeling and computer simulation. These simulation studies have been successful in reproducing many phenomena observed in animal experiments, for example the minimal block frequency, the influence of temperature on minimal block frequency, and the relationship between axon diameter and block threshold (Tai et al., 2005a,b, 2009a,b, 2011; Zhang et al., 2006; Bhadra et al., 2007; Liu et al., 2009; Ackermann et al., 2011). The newly discovered non-monotonic relationship between block threshold and stimulation frequency was also successfully reproduced in our recent simulation study of unmyelinated axons (Zhao et al., 2014). This study indicates that the monotonic decrease in block threshold in unmyelinated axons at frequencies above 15 kHz is probably caused by a slightly (<1 μs in pulse width) non-symmetric waveform of the high-frequency stimulation, which constantly hyperpolarizes or depolarizes the axon as the frequency increases above 15 kHz.

Although our previous simulation study (Tai et al., 2011) of large (10–20 μm diameter) myelinated axons showed a monotonic increase in block threshold with stimulation frequency up to 100 kHz, the effect of a non-symmetric waveform on the block threshold was not investigated. Based on our recent simulation study of unmyelinated axons (Zhao et al., 2014), we hypothesize that a non-symmetric waveform can also cause a decrease in block threshold as the frequency increases above a certain level in myelinated axons. It is known that the ion channel kinetics of myelinated axons is faster than unmyelinated axons (Hodgkin and Huxley, 1952; Frankenhaeuser, 1960). Therefore, it is reasonable to expect that a higher frequency would be required in myelinated axons than in unmyelinated axons in order to cause a constant hyperpolarization/depolarization by a non-symmetric stimulation waveform.

In this study we employed a myelinated axonal model (Frankenhaeuser–Huxley model) (Frankenhaeuser and Huxley, 1964; Rattay, 1989; Rattay and Aberham, 1993) to simulate high-frequency nerve block and to determine: (1) if a decrease in block threshold can be produced by high-frequency stimulation of a non-symmetric waveform; (2) At what frequency the decrease in block threshold can occur; (3) what happens to the sodium and potassium channels when this decrease in block threshold occurs. Understanding the mechanisms of nerve conduction block induced by high-frequency biphasic electrical stimulation will be very useful in developing new nerve blocking methods, optimizing stimulation parameters, or improving the efficacy of blocking nerves in different clinical applications.

## Methods

**Figure 1:**
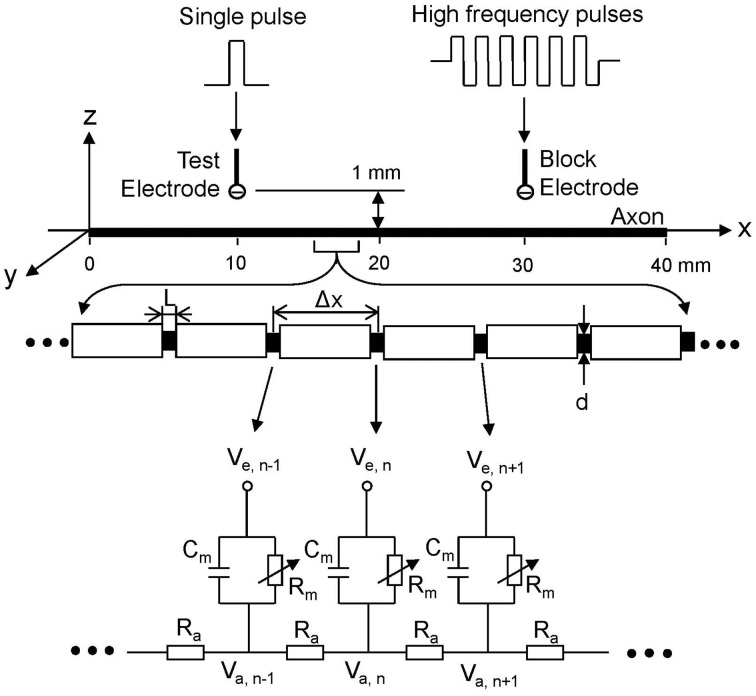
**Myelinated axonal model used to simulate conduction block induced by high-frequency biphasic electrical current**. The inter-node length Δ*x* = 100 d; d is the axon diameter. L is the nodal length. Each node is modeled by a resistance-capacitance circuit based on the FH model. R_a_, inter-nodal axoplasmic resistance; R_m_, nodal membrane resistance; C_m_, nodal membrane capacitance; V_a,n_, intracellular potential at the nth node; V_*e,n*_, extracellular potential at the nth node.

The myelinated axon model used in our study is showed in Figure 1. A 40 mm long, myelinated axon is modeled with the inter-node length Δ*x* = 100 d (d is the myelinated axon diameter). Each node (nodal length: *L* = 2.5 μm) is modeled by a membrane capacitance (C_m_) and a variable membrane resistance (R_m_). The ionic currents passing through the variable membrane resistance are described by Frankenhaeuser–Huxley equations (Frankenhaeuser and Huxley, 1964; Rattay, 1989; Rattay and Aberham, 1993). Two monopolar point electrodes (with the indifferent electrode at infinity) are placed at 1 mm distance from the axon. One is the block electrode at the 30 mm location along the axon, where the high frequency biphasic stimulation without inter-pulse interval is delivered (Figure 1). The other is the test electrode at 10 mm location, which delivers a uniphasic single pulse (pulse width 0.1 ms and intensity 0.5–2 mA) to evoke an action potential and test whether this action potential can propagate through the site of the block electrode. The test electrode is always the cathode (negative pulse), and the block electrode delivers biphasic pulses with the cathodal phase first.

We assume that the myelinated axon is in an infinite homogeneous medium (resistivity ρ_*e*_ = 300 Ωcm). After neglecting the small influence of the axon in the homogeneous medium, the extracellular potential *V_e, n_* at the n^th^ node along the axon can be described by:

$$V_{e,n}=\frac{\rho_{e}}{4\pi }\left[\frac{I_{block}(t)}{\sqrt{{\left(n\Delta x-x_{0}\right)}^{2}+z_{0}^{2}}}+\frac{I_{test}(t)}{\sqrt{{\left(n\Delta x-x_{1}\right)}^{2}+z_{1}^{2}}}\right]$$

where *I_block_*(*t*) is the high-frequency biphasic current delivered to the block electrode (at location *x*_0_ = 30 mm, *z*_0_ = 1 mm); *I_test_*(*t*) is the single test pulse delivered to the test electrode (at location *x*_1_ = 10 mm, *z*_1_ = 1 mm).

The change of the membrane potential *V_n_* at the n^th^ node of the myelinated axon is described by:

$$\begin{array}{l} \frac{dV_{n}}{dt}=[\frac{d\Delta x}{4\rho_{i}L}(\frac{V_{n-1}-2V_{n}+V_{n+1}}{\Delta x^{2}}\\ \;+\frac{V_{e,n-1}-2V_{e,n}+V_{e,n+1}}{\Delta x^{2}})-I_{i,n}]/c_{m} \end{array}$$

where *V_n_ = V_a, n_-V_e, n_-V_rest_*; *V_a, n_* is the intracellular potential at the n^th^ node; *V_e, n_* is the extracellular potential at the n^th^ node; *V_rest_* is the resting membrane potential; ρ_i_ is the resistivity of axoplasm (100 Ωcm); *c_m_* is the capacity of the membrane (2 *uF/cm^2^*); *I_i, n_* is the ionic current density at the n^th^ node described by Frankenhaeuser–Huxley equations (Frankenhaeuser and Huxley, 1964; Rattay, 1989; Rattay and Aberham, 1993; Zhang et al., 2006).

The myelinated axon model was solved by Runge-Kutta method (Boyce and Diprima, 1997) with a time step of 0.5 μs. The simulation was always started at initial condition *V*_n_ = 0. The intracellular potentials at the two end nodes of the modeled axon were always equal to the intracellular potentials of their closest neighbors, which implemented sealed boundary conditions (no longitudinal currents) at the two ends of the modeled axon. The block threshold current was determined with a resolution of 0.1 mA. The simulation was performed on a myelinated axon of diameter 2 μm with the temperature parameter set at 37°C (Rattay and Aberham, 1993).

## Results

### Conduction block by symmetric and non-symmetric biphasic stimulation waveforms

**Figure 2:**
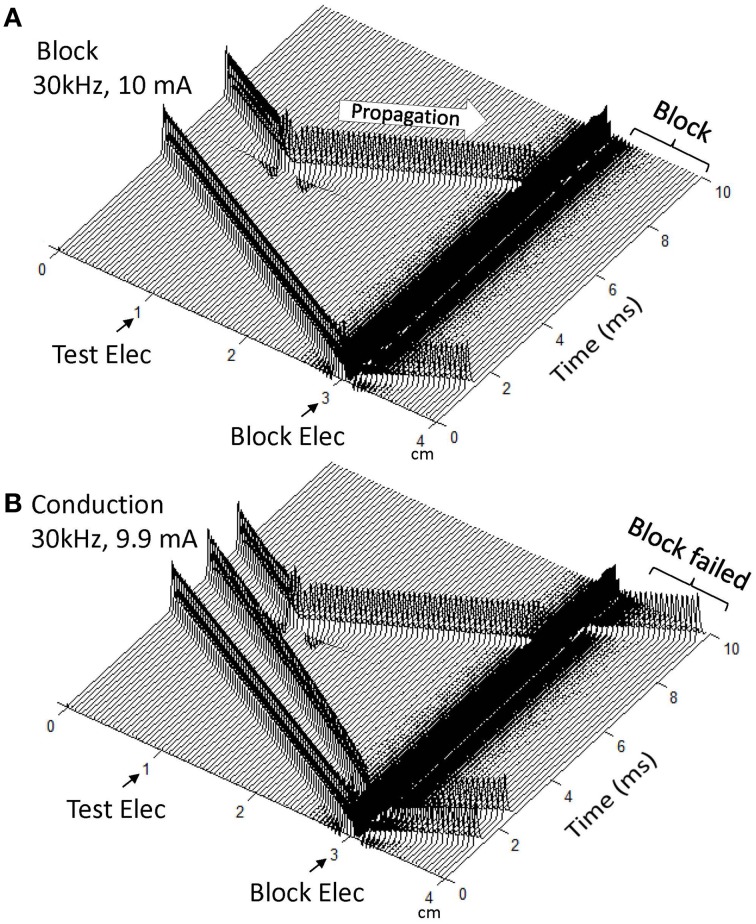
**Blocking the propagation of action potentials along a myelinated axon by high-frequency symmetric biphasic stimulation**. High-frequency (30 kHz) stimulation is continuously delivered at the block electrode, which initiated an initial action potential in **(A)** and two initial action potentials in **(B)**. Another action potential is initiated via the test electrode at 5 ms after starting the high-frequency stimulation, and propagates toward both ends of the axon. The 30 kHz stimulation blocks nerve conduction at the intensity of 10 mA **(A)**, but not at 9.9 mA **(B)**. Both symmetric and non-symmetric waveforms induced initial action potentials and axonal conduction block, which are dependent on stimulation frequency and intensity as shown previously [33]. The short arrows mark the locations of test and block electrodes along the axon. The white arrow indicates propagation of the action potential to the location of the 30 kHz blocking stimulation. Axon diameter: 2 μm.

Figure 2 shows that in a myelinated axon the Frankenhaeuser–Huxley model can successfully simulate the conduction block induced by high-frequency (30 kHz) symmetric biphasic stimulation. In Figure 2A the 30 kHz blocking stimulation (10 mA) generates an initial action potential propagating in both directions. At 5 ms after the start of blocking stimulation, the test electrode delivers a single pulse that generates another action potential propagating toward the block electrode (see the white arrow in Figure 2A). This action potential fails to propagate past the block electrode due to the presence of the high-frequency biphasic stimulation. However, at a lower stimulation intensity (9.9 mA in Figure 2B) the 30 kHz stimulation does not block nerve conduction and the action potential propagates through the site of the block electrode. Similar conduction block was also successfully simulated for non-symmetric biphasic stimulation waveforms where either the positive pulse is 1 or 2 μs longer than the negative pulse, or the reverse of this condition. Both symmetric and non-symmetric waveforms induced similar initial action potentials (see Figure 2), which was dependent on stimulation frequency and intensity as shown previously (Tai et al., 2009b).

**Figure 3:**
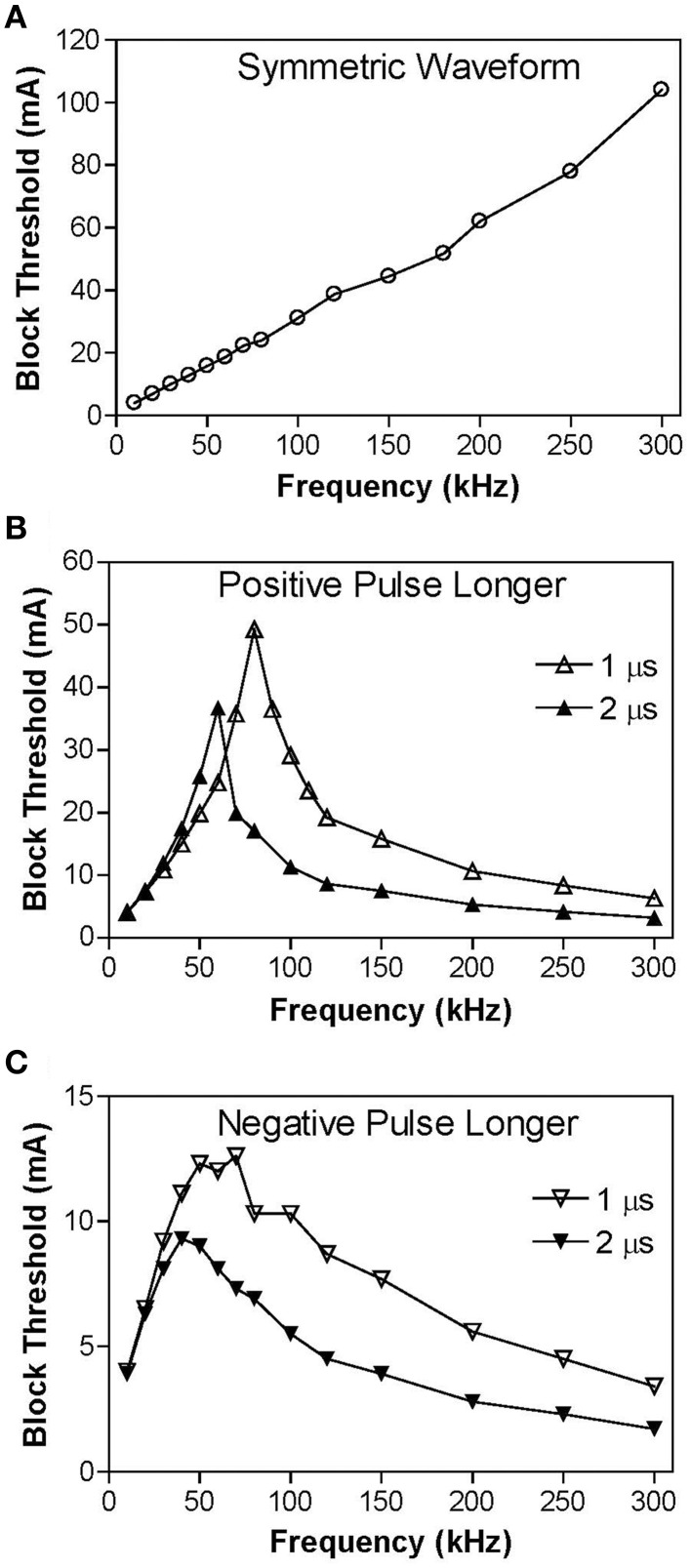
**The threshold intensity to block nerve conduction changes with the stimulation frequency. (A)** For the symmetric waveform, the block threshold monotonically increases as the frequency increases. **(B)** If the positive pulse is longer (1 or 2 μs), the block threshold peaks at 60–80 kHz and then gradually decreases as the frequency increases. **(C)** If the negative pulse is longer (1 or 2 μs), the block threshold peaks at 40–70 kHz. Axon diameter: 2 μm.

Figure 3 shows the intensity thresholds for inducing conduction block at different frequencies (10–300 kHz) for a myelinated axon of 2 μm diameter. For the symmetric biphasic waveform (Figure 3A), the block threshold monotonically increases as the stimulation frequency increases. However, if the biphasic waveform is not symmetric (Figures 3B,C), the block threshold increases initially and then decreases with increasing stimulation frequency, showing a non-monotonic relationship between block threshold and stimulation frequency. If the positive pulse is 1–2 μs longer than the negative pulse, the block threshold peaks between 60 kHz and 80 kHz (Figure 3B) with a value about two times the threshold for symmetric waveform. However, if the negative pulse is 1–2 μs longer than the positive pulse, the block threshold peaks at a frequency of 40–70 kHz (Figure 3C) with a value about 30–50% less than the threshold for symmetric waveform.

### Mechanisms of the conduction block by symmetric and non-symmetric waveforms

**Figure 4:**
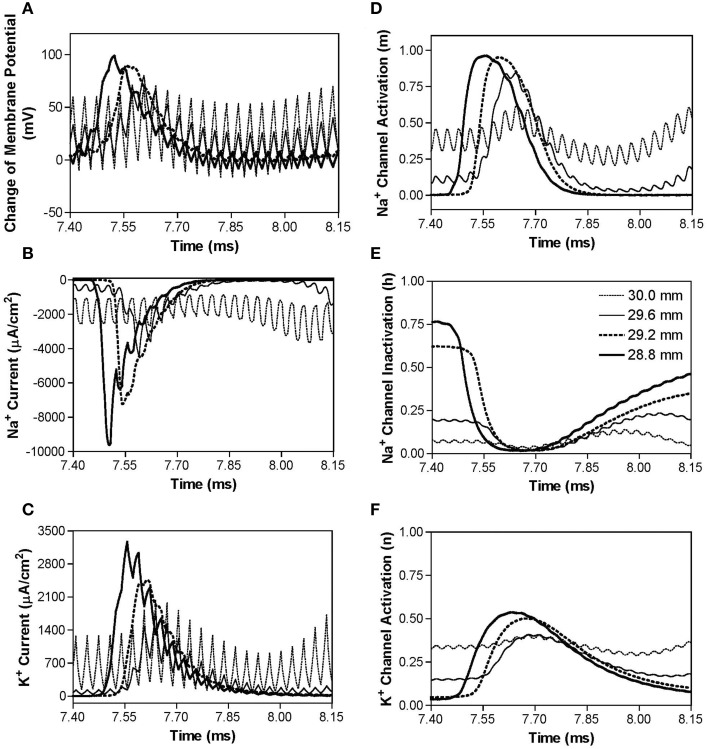
**The changes in membrane potential, ionic currents, and activation/inactivation of ion channels near the block electrode when conduction block occurs as shown in Figure 2A during stimulation with a symmetric waveform**. The legends in **(E)** indicate the locations along the axon. The location at 30.0 mm is under the block electrode. **(A)** Change in membrane potential, **(B)** Na^+^ current, **(C)** K^+^ current, **(D)** Na^+^ channel activation, **(E)** Na^+^ channel inactivation, **(F)** K^+^ channel activation. Symmetric stimulation waveform: 30 kHz, 10 mA. Axon diameter: 2 μm. Abscissa: time in ms after the start of blocking stimulation.

Figure 4 shows the same simulation as in Figure 2A for the 30 kHz symmetric biphasic waveform but including more detailed information for the four axon nodes at distances of 0–1.2 mm from the block electrode (the location at 30.0 mm is under the block electrode). Figures 4A–C show the action potential, sodium current, and potassium current at different locations approaching the block electrode. This action potential propagation is disrupted at the location (30.0 mm) under the block electrode, where axon membrane potential is oscillating with large pulsed sodium and potassium currents. The behavior of the membrane potential and ionic currents can be further explained by the activation/inactivation of the sodium and potassium channels as shown in Figures 4D–F. As the action potential propagates toward the block electrode, the activation (m) of sodium channels also changes at each location and becomes almost constant (about 0.4) at the location under the block electrode (Figure 4D). Meanwhile, the inactivation of sodium channels is kept at a low value (about 0.1) under the block electrode (Figure 4E). The combination of activation and inactivation of sodium channels (Figures 4D,E) determines that the sodium channel becomes constantly open and results in an oscillating inward sodium current under the block electrode (Figure 4B). Therefore, the sodium channels are never completely blocked when conduction block occurs. However, potassium channels are constantly activated at this location (Figure 4F), resulting in a large oscillating outward potassium current (Figure 4C). This large outward potassium current opposes the large inward sodium current, causing the membrane under the block electrode to become un-excitable leading to the block of action potential conduction. This blocking mechanism is observed for the symmetric waveform in the frequency range of 20–300 kHz (Figure 3A). Our previous simulation study (Zhang et al., 2006) has shown that at frequency range of 5–10 kHz the potassium channel but not the sodium channel is constantly open, which causes the conduction block.

**Figure 5:**
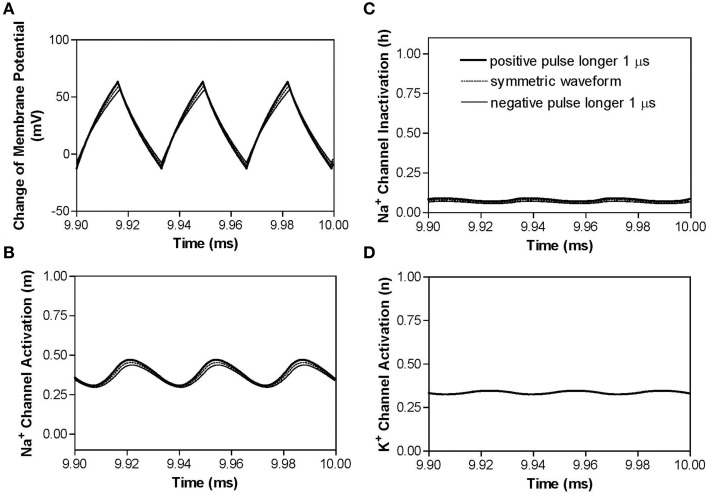
**The effects of non-symmetric waveforms on membrane potential and activation/inactivation of ion channels under the block electrode when stimulation frequency is 30 kHz**. The legends in **(C)** indicate the types of waveforms: symmetric and non-symmetric with a 1 μs difference in pulse width between the positive and negative pulses. **(A)** Change of membrane potential, **(B)** Na^+^ channel activation, **(C)** Na^+^ channel inactivation, **(D)** K^+^ channel activation. Stimulation waveforms: 30 kHz at block threshold intensities. Axon diameter: 2 μm. Abscissa: time in ms after the start of blocking stimulation.

Similar blocking mechanisms are also observed for non-symmetric waveforms at frequencies below the peak block threshold frequency (Figures 3B,C). Figure 5 shows that at 30 kHz the symmetric and non-symmetric waveforms produce almost the same oscillating membrane potential (Figure 5A) and very similar ion channel activation/inactivation (Figures 5B–D). It is also worth noting that the 1 μs difference between the positive and negative pulses does not change potassium channel activation (Figure 5D).

**Figure 6:**
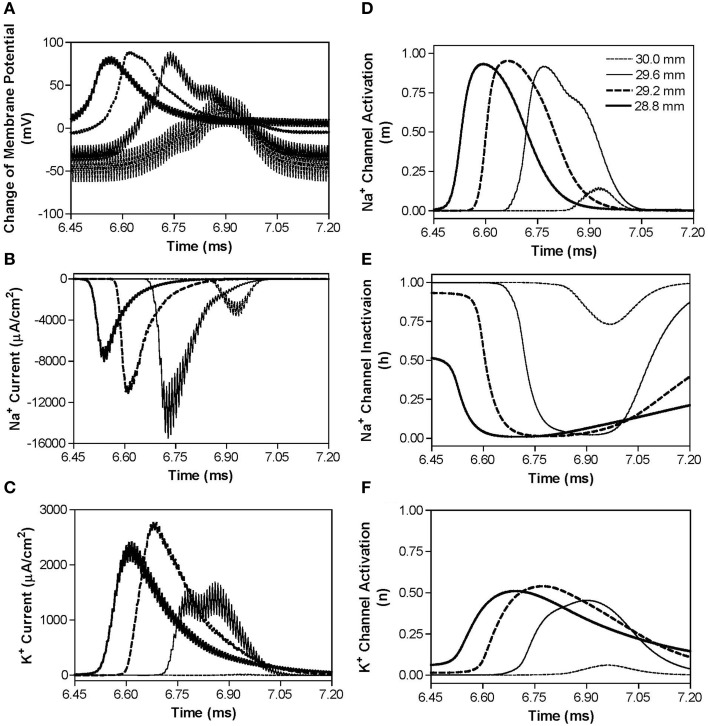
**The changes in membrane potential, ionic currents, and activation/inactivation of ion channels near the block electrode when conduction block is induced by a 120 kHz non-symmetric waveform with the positive pulse 1 μs longer than the negative pulse**. The legends in **(D)** indicate the locations along the axon. The location at 30.0 mm is under the block electrode. **(A)** Change in membrane potential, **(B)** Na^+^ current, **(C)** K^+^ current, **(D)** Na^+^ channel activation, **(E)** Na^+^ channel inactivation, **(F)** K^+^ channel activation. Non-symmetric stimulation waveform: 120 kHz, 19.2 mA. Axon diameter: 2 μm. Abscissa: time in ms after the start of blocking stimulation.

In order to understand why the block threshold with the non-symmetric waveform starts to decrease at frequencies above the peak block threshold frequency (Figures 3B,C), we further investigated the changes in membrane potential, ionic currents, and activation/inactivation of the sodium and potassium channels at frequencies between 50 and 300 kHz. Figure 6 shows the conduction block by the 120 kHz non-symmetric waveform with a positive pulse 1 μs longer than the negative pulse. Action potential propagation is completely abolished at the location (30.0 mm) under the block electrode, where the axon membrane is hyperpolarized to about −120 mV [(−50 mV) + (−70 mV resting potential), see Figure 6A]. This hyperpolarization is caused by the accumulative effect of 1 μs longer positive pulses, which significantly deactivates both sodium and potassium channels (Figures 6D,F), dramatically reduces sodium current (Figure 6B), and eliminates potassium currents (Figure 6C) thereby resulting in a conduction block at the location (30.0 mm) under the block electrode. Meanwhile, inactivation (h) of sodium channels is minimal (~1) under the block electrode (Figure 6E). The same blocking mechanism is observed at frequencies greater than 60–80 kHz for non-symmetric waveforms with the positive pulse 1–2 μs longer than the negative pulse (Figure 3B). As the frequency is increased, the accumulation of positive charges due to the longer positive pulses is greater and produces the same level of hyperpolarization at a lower stimulus intensity. Therefore, the block threshold decreases as the frequency increases (Figure 3B).

**Figure 7:**
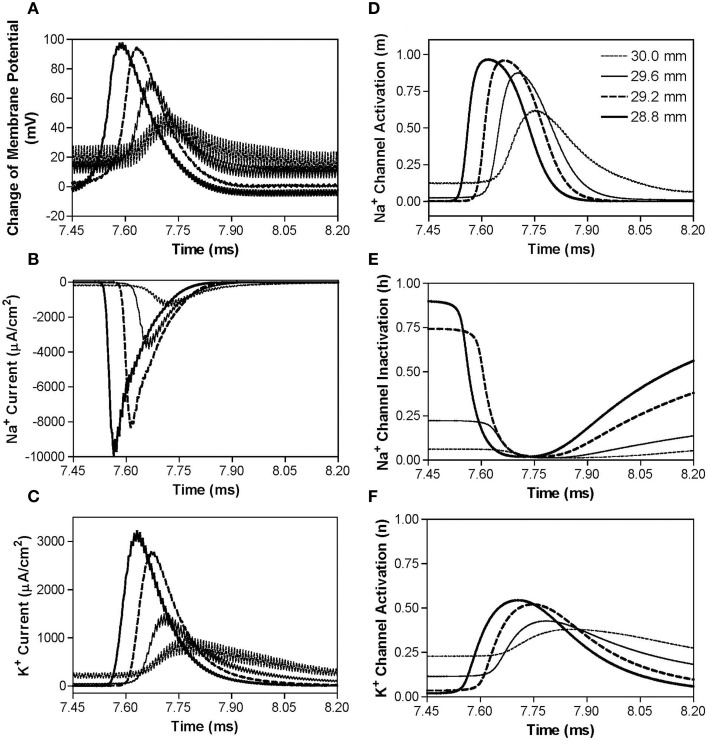
**The changes in membrane potential, ionic currents, and activation/inactivation of ion channels near the block electrode when conduction block is induced by a 120 kHz non-symmetric waveform with the negative pulse 1 μs longer than the positive pulse**. The legends in **(D)** indicate the locations along the axon. The location at 30.0 mm is under the block electrode. **(A)** Change in membrane potential, **(B)** Na^+^ current, **(C)** K^+^ current, **(D)** Na^+^ channel activation, **(E)** Na^+^ channel inactivation, **(F)** K^+^ channel activation. Non-symmetric stimulation waveform: 120 kHz, 8.7 mA. Axon diameter: 2 μm. Abscissa: time in ms after the start of blocking stimulation.

However, if the non-symmetric waveform has a longer negative pulse (1–2 μs longer), it generates a constant depolarization under the block electrode instead of a hyperpolarization when stimulation frequency increases higher than 40–70 kHz (Figure 3C). The non-symmetric waveform with the negative pulse 1 μs longer than the positive pulse produces a constant depolarization about 20 mV at the blocking electrode (Figure 7A), which causes a significant inactivation of sodium channels (Figure 7E) resulting in very small sodium current (Figure 7B) during stimulation thereby a conduction block. The accumulation of negative charges due to longer negative pulses is greater for a higher frequency, thereby producing the same level of depolarization at a lower block threshold (Figure 3C). It is worth noting that symmetric waveform can induce pulsed inward sodium currents during the stimulation (Figure 4B), while non-symmetric waveforms cannot induce pulsed inward sodium currents either due to sodium channel deactivation by a constant hyperpolarization (Figures 6A,B) or sodium channel inactivation by a constant depolarization (Figures 7A,B).

## Discussion

This study using the Frankenhaeuser–Huxley axonal model successfully simulated nerve conduction block in myelinated axons during high-frequency (10–300 kHz) biphasic electrical stimulation (Figure 2). It predicted a monotonic relationship between block threshold and stimulation frequency for a symmetric waveform (Figure 3A) and a non-monotonic relationship for non-symmetric waveforms (Figures 3B,C). The results reveal that the symmetric waveform causes conduction block by constantly activating both sodium and potassium channels (Figure 4) at frequencies of 20–300 kHz, while the non-symmetric waveforms share the same blocking mechanism as the symmetric waveform from 20 kHz up to the peak threshold frequency (Figure 5). However, at the frequencies above the peak threshold frequency the non-symmetric waveforms cause either hyperpolarization (Figure 6, positive pulse longer) or depolarization (Figure 7, negative pulse longer) and thereby conduction block. These results have significant implications for future animal experiments and for clinical applications of the nerve block methods.

This study predicts in myelinated axons that the block threshold will reach a peak and then gradually decrease when the stimulation frequency increases above approximately 50 kHz for non-symmetric waveforms (Figure 3). A similar non-monotonic block response has been observed in unmyelinated axons of sea slugs and frogs with the block threshold peaks ranging between 12 and 15 kHz (Joseph and Butera, 2009, 2011) and in our recent simulation study of unmyelinated axons (Zhao et al., 2014). Previous animal studies that examined block of myelinated axons (Bhadra and Kilgore, 2005; Gaunt and Prochazka, 2009; Joseph and Butera, 2011) only tested frequencies up to 50 kHz and showed a monotonic increase in block threshold as the frequency increases same as our simulation results at frequencies below 50 kHz (Figure 3). The results in this simulation study further suggest that additional animal studies should be conducted to examine higher frequencies (50–300 kHz) in myelinated axons and to confirm the different block responses for symmetric and non-symmetric waveforms.

This study emphasizes the importance of using a symmetric biphasic waveform for high-frequency nerve block of myelinated axons, especially when the frequency is above 50 kHz. The small difference of 1 μs between the positive and negative pulses (less than 19% difference in pulse width) may not make a difference in nerve block at frequencies below 50 kHz (Figures 4, 5), but can make a significant difference at frequencies of 50–300 kHz (19–50% difference in pulse width) causing a decrease in block threshold (Figures 3B,C) by constantly hyperpolarizing (Figure 6) or depolarizing (Figure 7) the axonal membrane. The net effect of the non-symmetric waveform on axonal conduction is equivalent to that caused by direct current (DC). The non-symmetric waveform with a longer positive (or negative) pulse blocks nerve conduction by inducing a constant hyperpolarization (or depolarization) of the axon membrane, which is similar to the nerve conduction block induced by an anodal (or cathodal) DC (Tai et al., 2009a). It is known that DC can damage nerves during long-term application due to the accumulation of electrical charges that can cause irreversible chemical reactions. Electrical charges could accumulate more rapidly when the stimulation frequency is high (such as >50 kHz, see Figures 6, 7) even with a very small difference (such as 1 μs) between the durations of the positive and negative pulses of the non-symmetric waveform. Therefore, the results from this simulation study suggest that waveform symmetry needs to be carefully examined when the high-frequency biphasic stimulation is to be used in clinical applications at a frequency greater than 50 kHz. Recent advances in electronic design provide the engineering methods and tools to generate a symmetric waveform with a very high accuracy (Sit and Sarpeshkar, 2007; Nag et al., 2013).

Although a high-pass filter could be used to remove the DC component of a non-symmetric waveform and make the waveform be charge-balanced, the filtered waveform will still be non-symmetric if the positive and negative pulse widths are different. Currently, no study has investigated nerve responses to a high frequency, biphasic, charge-balanced, non-symmetric stimulation waveform. It is unknown if nerve block can be induced by this type of stimulation waveform, because the nerve response is dependent on not only the total stimulation charge but also the time course of the stimulation waveform. Additional investigations by computer simulation and/or animal studies are certainly warranted.

This study and our previous studies (Zhang et al., 2006; Tai et al., 2011) using the myelinated axonal model (Frankenhaeuser–Huxley model) have revealed several different blocking mechanisms for different stimulation frequencies. These studies indicate that the kinetics of ion channel gating play a major role in the conduction block induced by high-frequency biphasic electrical stimulation. The kinetics of the potassium channel are slow compared to the sodium channel (Hodgkin and Huxley, 1952; Frankenhaeuser, 1960), and therefore this channel does not follow high-frequencies very well. Thus, the potassium channel becomes constantly open as the frequency increases to the minimal blocking frequency of about 4 kHz (Zhang et al., 2006; Liu et al., 2009). This potassium channel opening mechanism governs the monotonic increase in block threshold at the frequency range of 4–10 kHz (Zhang et al., 2006; Liu et al., 2009). As the frequency increases further (>20 kHz), it saturates the faster kinetics of the sodium channel causing the channel to be constantly open (Figures 4D, 5B) and lose its ability to regulate sodium current during action potential generation (Figure 4B) resulting in a conduction block (Figure 4A) (Tai et al., 2011). This sodium channel opening mechanism governs the monotonic increase in block threshold from 20 kHz to 300 kHz for a symmetric waveform (Figure 3A), but only to the frequency at which the block threshold peaks for non-symmetric waveforms (Figures 3B,C). Further increasing the stimulation frequency above the peak threshold frequency will cause either hyperpolarization (Figure 6) or depolarization (Figure 7) by the non-symmetric waveforms, which is responsible for the monotonic decrease in block threshold (Figure 3). The ion channel kinetic mechanisms are supported by evidence from animal studies indicating that the minimal blocking frequency is about 4 kHz (Reboul and Rosenblueth, 1939; Rosenblueth and Reboul, 1939; Bowman and McNeal, 1986) and that the block threshold monotonically increases in the frequency range of 4–50 kHz (Bhadra and Kilgore, 2005; Gaunt and Prochazka, 2009; Joseph and Butera, 2011). However, these ion channel kinetic mechanisms revealed by model analysis still need to be confirmed directly by animal studies in the future.

This study used the Frankenhaeuser–Huxley axonal model that has fixed parameters independent of stimulation frequency (Hodgkin and Huxley, 1952; Frankenhaeuser and Huxley, 1964; Rattay and Aberham, 1993). The stimulation amplitudes used in this study are well within the model's range, since they never cause the simulation to overflow and steady state responses were always achieved (Figures 2–7). Although the model parameters were obtained from voltage clamp experiments (low frequency response), the Frankenhaeuser–Huxley axonal model has been used successfully to simulate axonal responses for stimulation up to 50 kHz (Bromm, 1975; Reilly et al., 1985; Rattay, 1986). Our previous studies using the Frankenhaeuser–Huxley axonal model (Zhang et al., 2006; Tai et al., 2009b, 2011) have also successfully simulated high-frequency nerve block up to 100 kHz and reproduced many phenomena observed in animal experiments, for example the minimal block frequency, the influence of temperature on minimal block frequency, and the relationship between axon diameter and block threshold. However, whether the simulation results obtained in this study for stimulation frequency up to 300 kHz predict the real axonal block effect can only be confirmed by animal studies using myelinated nerve. This simulation study provides the rationale for and the expected results for future animal studies.

Nerve conduction block induced by high-frequency biphasic electrical stimulation has many potential applications in both clinical medicine and basic neuroscience research (Nashold et al., 1982; Tai et al., 2004; Camilleri et al., 2009; Waataja et al., 2011). Understanding the mechanisms underlying this type of nerve block could improve the design of new stimulation waveforms (Roth, 1994, 1995) and further promote clinical application (Leob, 1989; Song et al., 2008). Simulation analysis using computer models provides a tool to reveal the possible blocking mechanisms and may help to design new animal experiments to further improve the nerve blocking method.

### Conflict of interest statement

The authors declare that the research was conducted in the absence of any commercial or financial relationships that could be construed as a potential conflict of interest.
